# Supplementary material for: From sequence to enzyme mechanism using multi-label machine learning
Source: BMC Bioinformatics. 2014 May 19;15:150. doi: 10.1186/1471-2105-15-150 (PMC4229970; doi:10.1186/1471-2105-15-150)
Supplement: Additional file 2 — Java code of ml2db. Additional file ml2db_code.tar.gz contains the Java source code to run the multi-label machine learning experiments and save the results to database. The code’s Javadoc is included. [file 1471-2105-15-150-S2.zip › additional file 2/ml2db/ml2db/doc/main/ml2db/mulan/mulanarff/MulanDataSetManager.html]

MulanDataSetManager


JavaScript is disabled on your browser.


- Overview
- Package
- Class
- Use
- Tree
- Deprecated
- Index
- Help

- Prev Class
- Next Class

- Frames
- No Frames

- All Classes

- Summary:
- Nested |
- Field |
- Constr |
- Method

- Detail:
- Field |
- Constr |
- Method


main.ml2db.mulan.mulanarff

## Class MulanDataSetManager

- java.lang.Object
- - uk.ac.standrews.utils.main.database.Manager
  - - main.ml2db.weka.DataSetManager
    - - main.ml2db.mulan.mulanarff.MulanDataSetManager

- ---

    

  ```
  public class MulanDataSetManager
  extends DataSetManager
  ```

  This class generates a data set (instances, attributes, classes) in Mulan
  style (for multi-label machine learning). The main difference compared with
  the classic Weka ARFF, is that there are multiple classes and each is listed
  as a normal binary {0, 1} attribute. In order to distinguish the normal
  attributes from the class attributes, the class attributes values must also
  be specified in a separate Mulan XML.

  Version:
  :   7 May 2010

  Author:
  :   Luna De Ferrari ldf(at)st-andrews.ac.uk

- - ### Field Summary

    - ### Fields inherited from class main.ml2db.weka.DataSetManager

      `DUMMY_VALUE`
  - ### Constructor Summary

    Constructors

    Constructor and Description

    `MulanDataSetManager(MulanArff arff)`
  - ### Method Summary

    Methods

    | Modifier and Type | Method and Description |
    | --- | --- |
    | `MulanArff` | `getArffGenerator()` |
    | `MulanDataSetDbLoader` | `getDataLoader()` |
    | `MulanDataSetGenerator` | `getDataSetGenerator()` |

    - ### Methods inherited from class main.ml2db.weka.DataSetManager

      `fillArff, getArffProperties, getClassName, getDataSetChecker, getDataSetName, getDataSetWriter, initialisationIsCorrect, writeBaseArff`
    - ### Methods inherited from class java.lang.Object

      `equals, getClass, hashCode, notify, notifyAll, toString, wait, wait, wait`

- - ### Constructor Detail


    - #### MulanDataSetManager

      ```
      public MulanDataSetManager(MulanArff arff)
      ```
  - ### Method Detail


    - #### getArffGenerator

      ```
      public MulanArff getArffGenerator()
      ```

      **Overrides:**
      :   `getArffGenerator` in class `DataSetManager`


    - #### getDataLoader

      ```
      public MulanDataSetDbLoader getDataLoader()
      ```

      **Overrides:**
      :   `getDataLoader` in class `DataSetManager`


    - #### getDataSetGenerator

      ```
      public MulanDataSetGenerator getDataSetGenerator()
      ```

      **Overrides:**
      :   `getDataSetGenerator` in class `DataSetManager`


- Overview
- Package
- Class
- Use
- Tree
- Deprecated
- Index
- Help

- Prev Class
- Next Class

- Frames
- No Frames

- All Classes

- Summary:
- Nested |
- Field |
- Constr |
- Method

- Detail:
- Field |
- Constr |
- Method
